# 3-Nitropropionic Acid Induces Ovarian Oxidative Stress and Impairs Follicle in Mouse

**DOI:** 10.1371/journal.pone.0086589

**Published:** 2014-02-05

**Authors:** Jia-Qing Zhang, Ming Shen, Cheng-Cheng Zhu, Feng-Xiang Yu, Ze-Qun Liu, Nazim Ally, Shao-Chen Sun, Kui Li, Hong-Lin Liu

**Affiliations:** 1 Department of Animal Genetics, Breeding and Reproduction, College of Animal Science and Technology, Nanjing Agricultural University, Nanjing, China; 2 Key Laboratory of Farm Animal Genetic Resources and Germplasm Innovation of Ministry of Agriculture, Institute of Animal Science, Chinese Academy of Agricultural Sciences, Beijing, China; South China Agricultural University, China

## Abstract

Oxidative stress induces many serious reproductive diseases in female mammals and thus poses a serious threat to reproductive health. However, the relationship between reactive oxygen species (ROS)—induced oxidative stress and follicular development, oocyte and embryo quality is not clear. The aim of this study was to investigate the effect of ovarian oxidative stress on the health of follicle and oocyte development. Female ICR mice were dosed with 3-nitropropionic acid (3-NPA) at three different concentrations (6.25, 12.5 and 25 mg/kg) and saline (control) via continuous intraperitoneal injection for 7 days. The treatment with 12.5 mg/kg reduced the weight of mouse ovaries, and significantly increased ROS levels and the activities of antioxidant enzymes—total superoxide dismutase (T-SOD), glutathione peroxidase (GPx) and catalase (CAT) — in granulosa cells and ovarian tissues, but not in other tissues (brain, liver, kidney and spleen). The same treatment significantly increased the percentage of atretic large follicles, and reduced the number of large follicles, the number of ovulated oocytes, and the capacity for early embryonic development compared with controls. It also significantly decreased the ratio of *Bcl-2* to *Bax*, while causing an increase in the mRNA expression of (*SOD2*, *CAT* and *GP*_X_) and ROS levels in granulosa cells. Collectively, these data indicate that 3-NPA induces granulosa cell apoptosis, large follicle atresia, and an increase of ROS levels in the ovary. Therefore, we have established an in vivo model of ovarian oxidative stress for studying the mechanism of resulting damage induced by free radicals and for the screening of novel antioxidants.

## Introduction

Research over recent years has greatly improved the understanding of the important roles that reactive oxygen species (ROS) and oxidative stress play in mammalian female reproduction. Physiological levels of ROS control various signal transduction pathways in folliculogenesis, oocyte maturation and ovulation [1], [2]. Accumulating evidence shows that ROS provide vital signals for the initiation of atresia in antral follicles, and of apoptosis in the granulosa cells of antral follicles; this is triggered by various stimuli including pesticides, environmental chemicals, ionizing radiation, and gonadotropin withdrawal [3], [4].

The ovary is the source of oocytes and regulates the normal secretion of hormones in female mammals. ROS are produced within the follicle as a normal part of reproduction due to both internal and external factors. However, excessive generation of ROS causes oxidative stress, an important mediator of follicle and oocyte development: it can damage many important molecules and structures in oocytes and granulosa cells within the ovarian follicles, and can accelerate oocyte aging [5]–[7]. Moreover, oxidative stress is known to initiate or exacerbate pathological processes affecting female reproduction [8], [9].

However, the precise relationships between ROS-induced oxidative and female reproduction is poorly understood; processes such as follicular development, granulosa cell apoptosis, follicle atresia, ovulation number, fertilization and early embryonic development can not be investigated adequately in human systems, due to ethical research constraints. Therefore, mammalian models of ovarian oxidative stress are essential to enable studies that can address these important gaps.

3-nitropropionic acid (3-NPA) is an irreversible inhibitor of complex II in the mitochondria. It impairs cellular energy metabolism via inhibition of succinate dehydrogenase, which induces a reduction in ATP production and leads to oxidative stress [10]–[13]. Also, 3-NPA can cause the generation and release of ROS from mitochondria, mitochondrial DNA damage, and thus loss of mitochondrial function [14]–[16]. Release of excessive ROS from mitochondria is in fact the main source of cellular oxidative stress.. High ROS levels have been detected previously in brain tissue and follicular granulosa cells, when rats and mice were intraperitoneally injected with 3-NPA [13], [17]–[20]. However, there are few successful reports on the oxidative stress model specified in mouse ovary studies.

The aim of our study was to establish a novel animal model of ovarian oxidative stress, using the chemical 3-NPA to examine the relationship between ovarian oxidative stress and follicle and oocyte development capacity in the mouse.

## Materials and Methods

### Ethic statement

Animal care and use were conducted in accordance with the Animal Research Institute Committee guidelines of Nanjing Agricultural University, China. Mice were housed in a temperature-controlled room with proper darkness-light cycles, fed with a regular diet, and maintained under the care of the Laboratory Animal Unit (Permit number: SYXK (Su)2011-0036), Nanjing Agricultural University, China. This study was specifically approved by the Committee of Animal Research Institute, Nanjing Agricultural University, China.

### Animals and materials

Female, 4-week-old ICR mice were kept at a constant temperature (22–24°C) in a 12 hr light/dark cycle with unrestricted access to food and water. For injection, 3-NPA (Sigma, Location) was dissolved in normal saline and the pH was adjusted to 7.4 with sodium hydroxide. Mice were randomly divided into four treatment groups (n = 20): (i) injected with normal saline solution (control); (ii) treated with 6.25 mg/kg 3-NPA; (iii) treated with 12.5 mg/kg 3-NPA; (iv) treated with 25 mg/kg 3-NPA; The control and experimental groups were injected intraperitoneally with a 0.1 ml dose of the appropriate solution twice daily, at 12 h intervals (8:00 a.m. and 8:00 p.m.) for 7 days. Weight gain, body and organ weights of mice treated with different doses of 3-NPA (6.25, 12.5 and 25 mg/kg) and control were measured at 8 days.

### Sample collection

To determine the estrous stage of individuals at the time of sampling, we used vaginal smears for tissue collection [21]–[23].The mice used for tissue collection were killed by cervical dislocation under anesthesia induced by ether inhalation. Both ovaries were collected; one was used to isolate granulosa cells for ROS measurement, antioxidant enzyme analysis and molecular analysis, and the other was fixed in 4% paraformaldehyde and processed for later morphological analysis. The liver, kidney, spleen and brain were dissected out immediately, washed with normal saline, dried on a filter paper and weighted. The mass index (%) of internal organs was calculated as liver (kidney and spleen) mass/bodymass×100.

### Measurement of ROS

Ovaries (n = 5/group) were collected three and seven days following normal saline and 3-NPA (12.5 mg/kg) treatment. Granulosa cells and oocytes were obtained via puncture of the dominant ovarian follicle (>200 µm). ROS levels in granulosa cells and oocytes were measured using the GENMED Intracellular ROS Red Fluorescence Determination Kit and GENMED Cellular Superoxide Anion Colorimetric Quantitative Determination Kit (GENMED, Shanghai, China). Image J software was employed to analyze the optical density in each oocyte. ROS levels in ovary, brain, kidney, liver and spleen tissue were detected with the GENMED Tissue Superoxide Anion Colorimetric Quantitative Determination Kit. The oxidation of dihydroethidium bromide (DHE) or 2′, 7′-dichlorofluorescein diacetate (DCFH-DA) to their fluorescent products was used to estimate ROS levels. These procedures were performed according to the manufacturer's instructions.

### In situ terminal dUTP nick-end labeling (TUNEL) staining

After seven days, mice from different treatment (n = 5/group) were killed by cervical dislocation. The left ovaries were collected and fixed in 4% paraformaldehyde for the terminal deoxynucleotidyl transferase dUTP nick-end labeling (TUNEL) assay. The detailed procedure followed the protocol of In Situ Cell Death Detection Kit (Roche Applied Science, Shanghai, China). Briefly, 6 µm sections were cut from the ovaries embedded in paraffin, and were placed on to glass slides. After deparafinization and rehydration, the ovary sections were incubated with proteinase-K (200 µg/ml) for 15 min at 37°C and rinsed with phosphate-buffered saline (PBS) for 20 min. The sections were then incubated for 10 min at 4°C in a humidified chamber with 0.1% Triton X–100, washed for 20 min with PBS, terminal deoxynucleotidyl transferase (TdT) for 1 h at 37°C, and stained with 4′, 6-diamidino-2-phenylindole (DAPI).We used a laser scanning confocal microscope (Zeiss, Oberkochen, Germany) to obtain photographic images of the sections. TUNEL positive follicles were counted in sections viewed across the maximum diameter of each ovary.

### Histological evaluation of follicles

Right ovaries were embedded in paraffin after a 12-h fixation in 4% paraformaldehyde. They were then serially sectioned (6 µm), mounted on glass slides, and stained with haematoxylin and eosin for morphometric analysis. Ovarian follicles were counted according to established methods [24]. Briefly, every fifth ovary section was scanned under a dot Slide-digital virtual microscope, and the number of small, medium, and large, follicles in the entire section was counted. To avoid multiple counts of the same follicle, only those with a visible oocyte nucleus were included. Since oocyte nuclei measured between 20–30 µm in diameter, counting every fifth section of the ovary ensured a distance of 30 µm between analysed sections, minimizing the chance of multiple counts of the same ovarian follicle. The following follicle classification [25], [26] was used: Type 1, small follicles (Pedersen and Peters Types 1–3b): an isolated oocyte or one layer of flattened granulosa cells surrounding the oocyte; Type 2, medium follicles (Pedersen and Peters Types 4–5b): an oocyte surrounded by multilayered cuboidal granulosa cells, with no visible antrum; Type 3, large follicles (Pedersen and Peters Types 6–8): an oocyte surrounded by multiple layers of cuboidal granulosa cells and containing one or more antral spaces, cumulus oophorus, with theca layer may also have been evident. Atretic follicles were identified using standard methods [26], [27]; follicles were considered atretic if they contained more than 10 pyknotic nuclei, disorganized granulosa, a degenerating oocyte, or a fragmented oocyte nucleus.

### Assay of antioxidant enzyme activity

After 3-NPA (12.5 mg/kg) treatment for seven days, different tissues (ovary, brain, spleen, liver and kidney) were collected. Separately, tissue from each of the five sampled organs (ovary, brain, spleen, liver and kidney) was homogenized in cold saline to prepare for the assay for activity of antioxidant enzymes. The activities of three enzymes—T-SOD, GPx and CAT—were determined using commercial kits (Nanjing Jiancheng Bioengineering Institute, Nanjing, China). (1) T-SOD activity was assayed using the xanthine/xanthine oxidase method based on the production of O^2−^ anions. (2) GP_X_ activity was estimated based on its catalyzation by the oxidation of reduced glutathione in the presence of cumene hydroperoxide. The generation of nicotinamide adenine dinucleotide phosphate was measured spectrophotometrically at 340 nm. (3) CAT activity was measured by analyzing the rate at which it caused the decomposition of H_2_O_2_ at 240 nm, the substrate of the enzyme contained in various tissue samples. Activities of T-SOD and GP_X_ are expressed as units per milligrams of protein (U/mg protein). The activity of CAT is expressed as (U/g protein).

### Quantitative real time polymerase chain reaction (qPCR)

Total RNA was extracted from granulosa cells and other tissues (brain, spleen, liver and kidney)) using TRIZOL (Invitrogen). RNA concentrations were measured with spectrophotometry (OD260/OD280), and RNA integrity was evaluated using electrophoresis using 1% formaldehyde denaturing agarose gel. Quantification of each mRNA level was performed with a real-time polymerase chain reaction (RT-PCR) using a LightCycler (ABI) and real-time PCR Premix with SYBR Green (TaKaRa) in a reaction volume of 20 µl. Thermal cycling was performed for 40 cycles; each cycle consisted of 5 seconds of denaturation at 95°C, with 31 seconds at each annealing temperature.GAPDH was used as an internal control and each sample was run in triplicate. Primers for relevant apoptosis genes and antioxidant enzyme genes (Bax, Bcl-2, SOD2, CAT and GP_X_) were designed by the Primer 5 software. Primer sequences are shown in supplemental Table S1.

### In vivo fertilization and culture of zygotes

After 3-NPA (12.5 mg/kg) administration for five days, mice (n = 5/group) were superovulated with intraperitoneal injections of pregnant mare serum gonadotropin (PMSG), followed with human chorionic gonadotropin (hCG) 48 h later. Eggs were obtained from the oviducts 20 h after hCG injection, and then fertilized in vitro [28]. The number of ovulated oocytes was counted, and the rates of fertilization, cleavage, and blastocyst formation were observed under an inverted phase-contrast microscope.

### Hormone assays

Mice that were in the same estrus cycle were treated with normal saline and 3-NPA (12.5 mg/kg) for seven days. Approximately, 1 ml blood was removed from each animal while killing, allowed to clot at room temperature for 20 min, and then centrifuged at 6000 g for 20 min to extract serum. Serum samples were stored at −80°C until processed for FSH, LH,E2 and P4 measurements using mouse ELISA kits according to the manufacturer's instructions (Nanjing Sen Beijia Biological Technology Co., Ltd., Nanjing, China). The limits of sensitivity of the assays were 0.5 IU/L for FSH, 100 ng/L for LH, 1 pmol/L for E2, 50 pmol/Lfor P.

### Reproductive performance

Mice (n = 81) were divided into two groups that were treated for seven days: a control group injected with normal saline, and an experimental group treated with 3-NPA (12.5 mg/kg). To induce ovulation, individuals were administered 5 IU of hCG intraperitoneally, 48 h after administration of 5 IU of PMSG. Subjects were then paired with male mice (one to one) overnight, and were examined for the presence of a vaginal plug in the following morning. The day on which a vaginal plug was observed was considered as gestation day (GD) 0.5. All timed-pregnant female mice were housed individually and euthanized on GD7.5, 12.5 and 18.The number of fetal mice carried by each subject in the experimental and control groups was counted.

### Behavioral assessment

#### Rota-rod test

All animal were evaluated for motor ability and balance by using the rotarod apparatus. The mice were given a prior training session before initialization of therapy to acclimate them to rotarod apparatus. Mice were placed on the rotating rod with a diameter of 7 cm (speed 20 rpm). The length of time on the rod was taken as the measure of competency. The cut off time as 300 s and each mouse performed three separate trials. The average results were recorded as fall of time. Comparisons were made between the control animals and 3-NPA (12.5 mg/kg) treatment animals. The difference in the fall off time from the rotating rod between the control and treated groups were taken as index of motor incoordination and balance [29].

#### Forced swimming test (FST)

Each mouse was placed individually in a glass cylinder (diameter 12 cm, height 24 cm) filled with water at a height of 12 cm. Water temperature was maintained at 23–25°C. The animal was forced to swim for 6 min once a day for 3 consecutive days. Animals were then allowed to return to their home cage. On the 4nd day, each mouse was placed again into the water and forced to swim for 6 min. The duration of immobility during the last 4 min was measured. The mouse was considered as immobile when it stopped struggling and moved only to remain floating in the water, keeping its head above water [30].

#### Tail suspension test (TST)

On the test day mice were moved from the housing colony room to the testing laboratory and allowed to adapt to the new environment for 1 h before testing. Mice were suspended on the edge of a shelf 60 cm above a table top by adhesive tape placed approximately 1 cm from the tip of the tail. A total of 6 min throughout the test, the duration of immobility during the last 4 min was measured. Mice were considered immobile only when they hung passively and completely motionless [31].

### Measurement of telomerase activity and average telomere length

Tissue samples were flash frozen in liquid nitrogen immediately after removal and stored at −80°C. For telomerase extraction approximately 30 mg of tissue was washed twice in ice-cold phosphate-buffered saline (PBS), and finally homogenized in about 150 ml of PBS. Homogenates were kept on ice for 30 min and were then centrifuged at 3000 rpm for 20 min at 4°C, and the supernatant was rapidly frozen and stored at −80°C until processed for telomerase (TE) activity using mouse TE ELISA kits according to the manufacturer's instructions (Nanjing Sen Beijia Biological Technology Co., Ltd., Nanjing, China). The limits of sensitivity of the assays were 0.8 IU/L for TE.

Genomic DNA was isolated according to standard procedures using the TIAN amp Genomic DNA Kit (Tian Gen Biotech (Beijing) CO., Ltd., Catalogue No.: DP304). Average telomere length was measured from total genomic mouse DNA by using a real-time quantitative PCR method previously described [32]–[35]. Standard curves were generated for telomere lengths and the single gene copy amplification reactions from a reference DNA sample serially diluted with Milli-Q water by 4-fold per dilution to produce five concentrations of DNA ranging from 160 to 0.625 ng/µL. Triplicate PCR reactions using 2 µ L of each DNA dilution were performed with SYBR Premix Ex Taq (DRR420A; TaKaRa) in a 20 µL reaction mixture according to the manufacturer protocol. Primers for telomeres and the single copy gene 36B4 were added to final concentrations of 0.3 µ M and 0.2 µ M, respectively. The primer sequences are

tel1, 5′-CGGTTTGTTTGGGTTTGGGTTTGGGTTTGGGTTTGGGTT-3′;

tel2, 5′-GGCTTGCCTTACCCTTACCCTTACCCTTACCCTTACCCT-3′

(sequences obtained from the published literature [36]);

36B4u, 5′-ACTGGTCTAGGACCCGAGAAG-3′;

36B4d, 5′-TCAATGGTGCCTCTGGAGATT-3′

(sequences obtained from the published literature [37]).

All PCRs were performed on the ABI 7300 (Applied Biosystems, USA). An automated thermocycler was used with reaction conditions set at 95°C for 10 min followed by 35 cycles of data collection at 95°C for 15 s and a 56°C anneal–extend step for 1 min for the telomere reaction, or 94°C for 10 s followed by 35 cycles of data collection at 95°C for 5 s and a 54°C anneal–extend step for 31 s for the 36B4 reaction. Real-time PCR was performed a minimum of 3 times for each sample, and the ratio of telomere: 36B4 was calculated. The average of these ratios was reported as the average telomere length ratio (ATLR).

### Statistical analysis

All data were analyzed using the software SPSS version 16.0 (SPSS Inc., Chicago, IL, USA). The mean number of small, medium and large follicles per ovary was calculated using ovaries from at least five different animals. Differences between the means were evaluated using a one-way ANOVA, with statistical significance assigned at *P*<0.05. When a significant result was observed, the Scheffe's test was used for post hoc analysis. Results are expressed as mean ± S.E.M.

## Results

### Effect of 3-NPA on weight gain, body and organ weights

**Figure 1:**
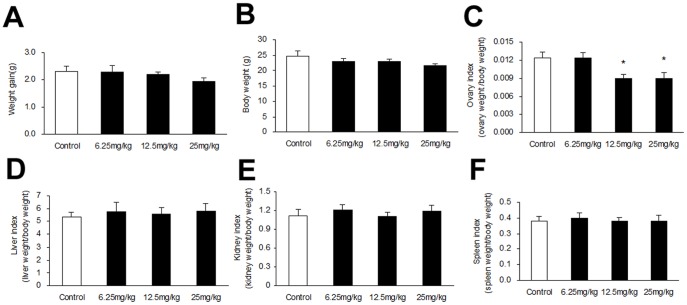
Effect of 3-NPA treatment at varying doses for 7 days on weight gain, body and organ weights. (A) Weight gain and (B) Body weight among groups. (C) Ovary, (D) Liver, (E) kidney and (F) spleen indices after 3-NPA injection. Bars represent means ± SEM (n = 10). * Significant difference from control, *P*<0.05.

Treatment with 3-NPA (25 mg/kg) resulted in a slight reduction in weight gain and body weight compared with control, yet there was no difference at lower concentrations of 3-NPA (Figure 1, A and B). Mice exposed to doses of 12.5 or 25 mg/kg displayed a significant reduction in ovary index, but there was no significant difference between mice treated with 6.25 mg/kg compared to the control (Figure 1C). Liver, kidney and spleen indices were not affected by the treatments (Figure 1, D, E and F).

### Effect of 3-NPA on ROS generation in different tissues

**Figure 2:**
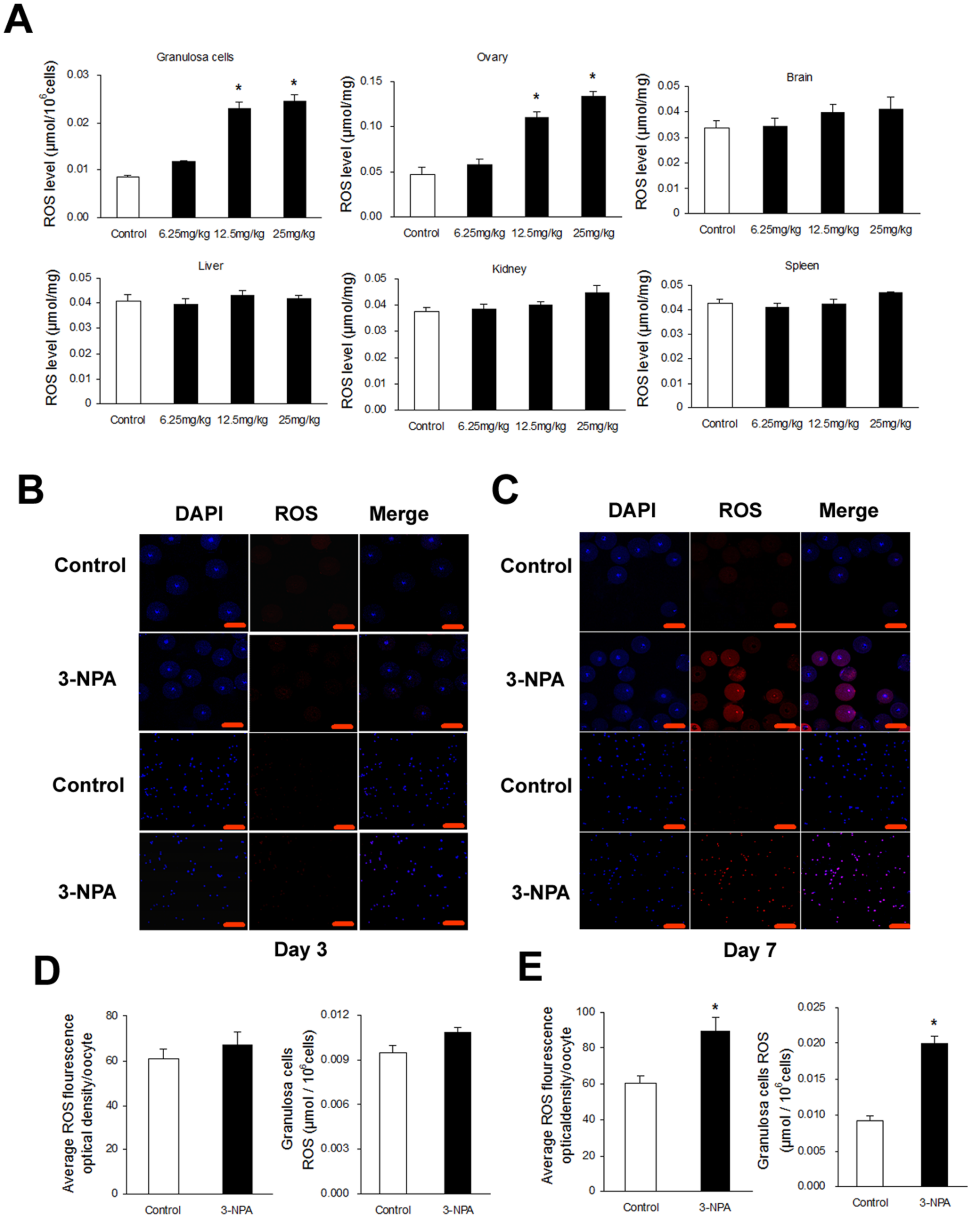
Effect of 3-NPA treatment over different time periods on ROS levels in different tissues. (A) Mice were intraperitoneally injected with saline versus 3-NPA (12.5 mg/kg) for 7 days. ROS levels in granulosa cells and other tissues were quantified by nitro blue tetrazolium (NBT) staining. (B and C) Mice were intraperitoneally injected with saline or 3-NPA (12.5 mg/kg) for 3 and 7 days, respectively. ROS levels in granulosa cells and oocytes were detected by dihydroethidium bromide fluorescence (red), and nuclei were counterstained with DAPI (blue). (D and E) ROS levels in granulosa cells were quantified by nitro blue tetrazolium (NBT) staining. Scale bars are 100 µm. Bars represent means ± SEM (n = 5); *significant difference from control, *P*<0.05.

Images obtained from granulosa cells and oocytes are shown in (Figure 2, B and C). ROS levels increased significantly in granulosa cells and ovarian tissues, but this did not increase in brain, kidney, liver and spleen tissue compared to the control group, for mice in the 12.5 mg/kg twice daily for 7 days treatment (Figure 2A), while treatment with the same dose for 3 days did not cause an increase (data not shown).

### Effect of 3-NPA on the activity of antioxidant enzymes and their mRNA level in different tissues

**Figure 3:**
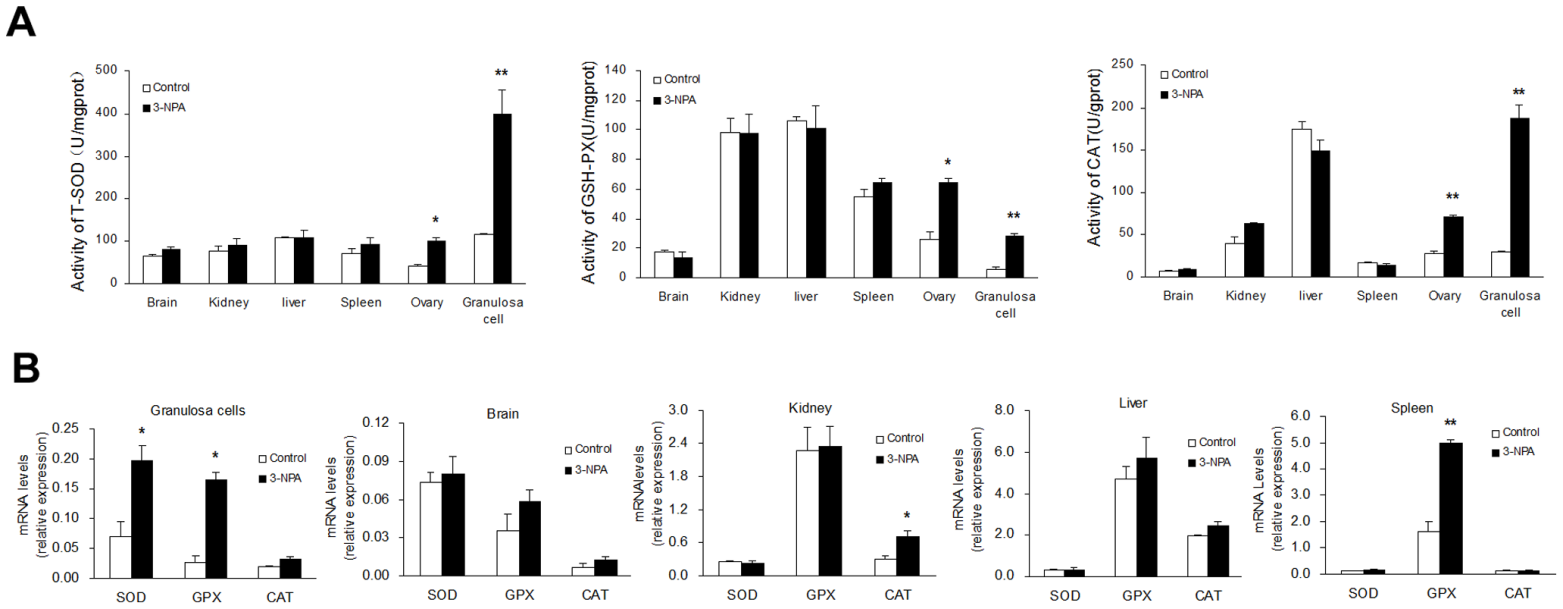
Effect of 3-NPA injection for 7 days on antioxidant enzymes activities and mRNA levels in different organ tissues. Mice were intraperitoneally injected with saline versus 3-NPA (12.5 mg/kg) for 7 days. (A) Antioxidant enzymes activities. (B) Relative expression levels of SOD2, GP_X_ and CAT. Data show the means ± SEM (n = 5). ^*^ *P*<0.05, ^**^ *P*<0.01 compared to control.

In granulosa cells and ovarian tissues, activities of the antioxidant enzymes (T-SOD, GPx and CAT) increased significantly in mice treated with 3-NPA (12.5 mg/kg). Enzyme activities within other tissues (brain, kidney, liver, and spleen) were not affected by treatment (Figure 3A).Quantitative PCR analysis showed that mRNA levels of *SOD2* and *GP_X_* in granulosa cells increased significantly after treatment, while *CAT* mRNA levels were similar to those of the control group (Figure 3B). *CAT* mRNA levels increased significantly in kidney tissue, but *SOD2* and *GP_X_* mRNA levels were not affected. In spleen tissue, *GP_X_* mRNA levels were increased significantly after treatment, while mRNA levels of *CAT* and *SOD2* did not change. There was no significant effect of 3-NPA injection on brain or liver tissue (Figure 3B).

### Effect of oxidative stress on apoptosis of granulosa cells in antral follicles

**Figure 4:**
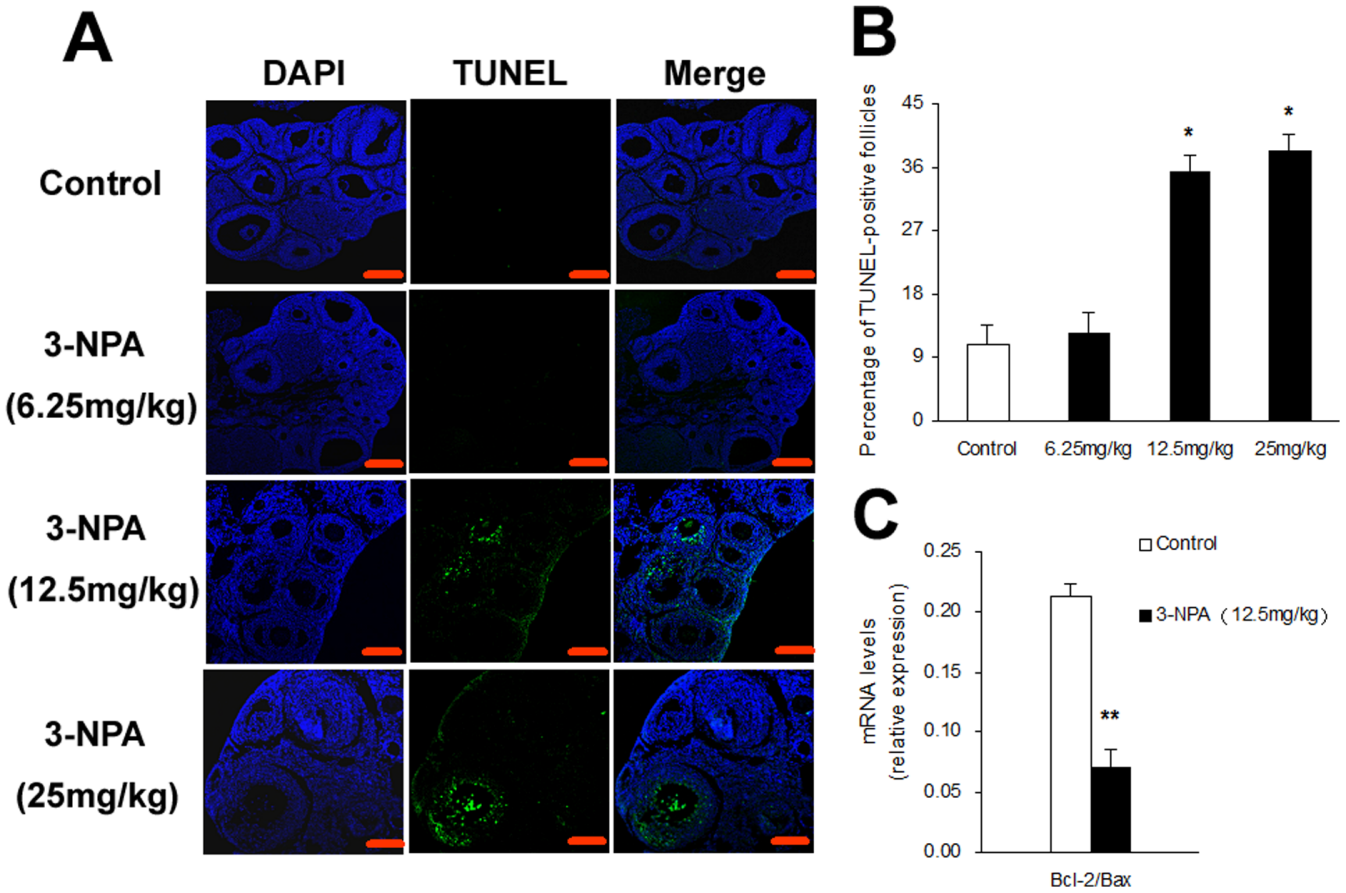
Effect of oxidative stress on granulosa cell apoptosis. Ovaries were collected after 3-NPA different doses treatment for 7 days (A) TUNEL staining of ovarian paraffin sections. Scale bars are 100 µm. (B) The percentage of TUNEL-positive follicles. (C) The relative expression levels of Bcl-2 and Bax in granulosa cells. Data show the means ± SEM (n = 5). ^*^ *P*<0.05, ^**^ *P*<0.01 compared to control.

Mice treated with 3-NPA for 7 days (12.5 or 25 mg/kg) displayed significantly higher levels of apoptosis in granulosa cells compared to those treated with the lowest dose and control groups (Figure 4A). The same two doses also resulted in a significant increase in the percentage of TUNEL-positive follicles (Figure 4B). Quantitative PCR analysis of the relative expression of Bcl-2 and Bax mRNA in granulosa cells showed that after treatment with 12.5 mg/kg for 7 days, the ratio of Bcl-2 levels to Bax levels was significantly decreased in granulosa cells compared with control (Figure 4C).

### Effect of oxidative stress on follicular development, atresia and oocyte development capacity

**Figure 5:**
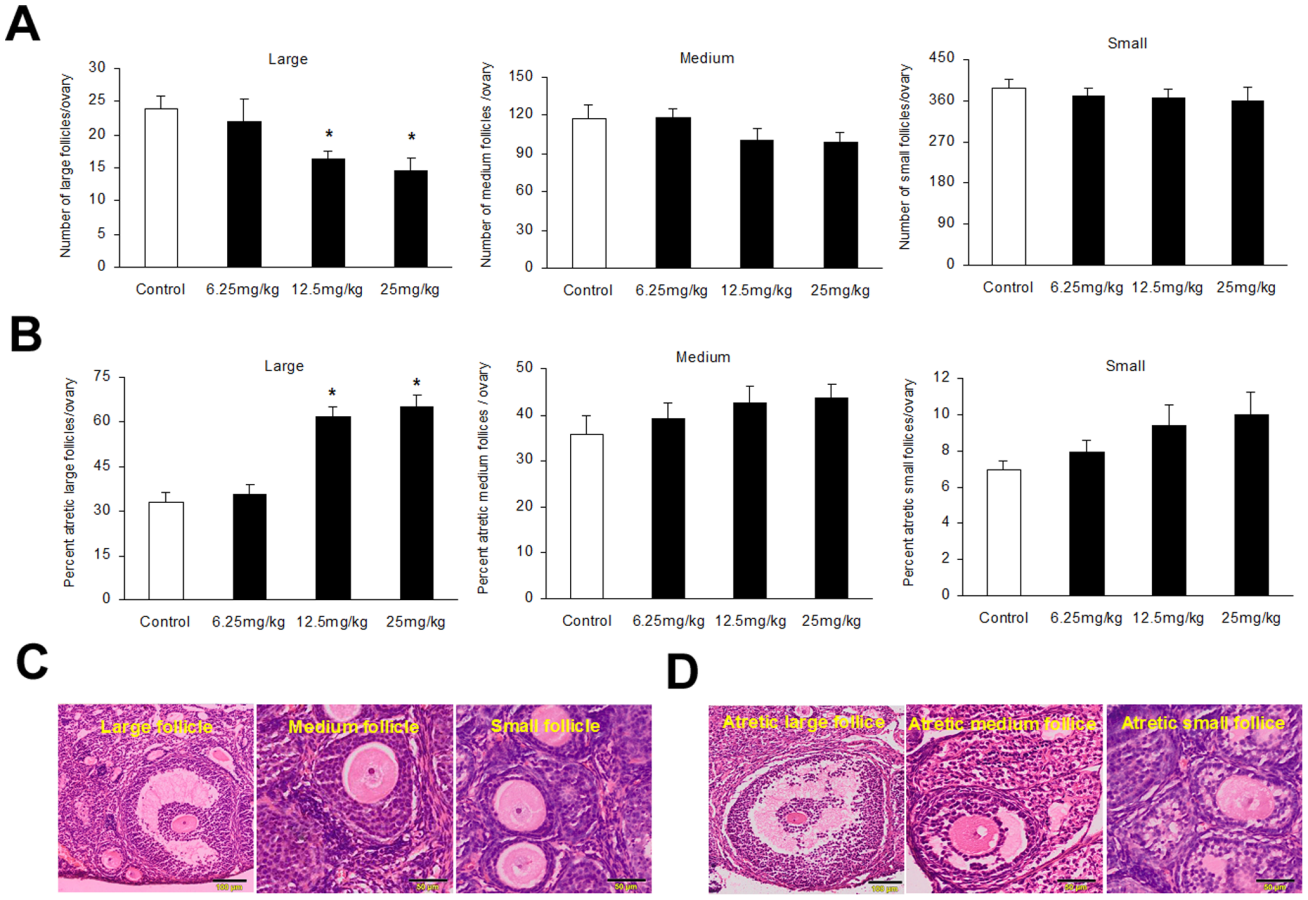
Effect of oxidative stress on number of follicles per ovary and histological analysis of follicles. (A) Number of small, medium and large follicles. (B) Percentage of atretic follicles (C) Representative photographs of haematoxylin and eosin-stained ovarian sections (6 µm) demonstrating healthy follicles. (D) Representative photographs of haematoxylin and eosin-stained ovarian sections (6 µm) demonstrating atretic follicles. Bars represent means ± SEM (n = 5); *significant difference from control, *P*<0.05.

Treatment with 3-NPA (12.5 or 25 mg/kg) resulted in a significant reduction in the number of large follicles in mouse ovaries, while the number of small and medium follicles did not change (Figure 5A). Those treatments also caused a significant increase in the percentage of atresia, but only in large follicles (Figure 5B).Representative photographs of haematoxylin and eosin-stained ovarian sections (6 µm) demonstrating representative classes of follicles are shown in (Figure 5C), along with the histological morphology of healthy follicle versus atretic follicles (Figure 5D).

**Figure 6:**
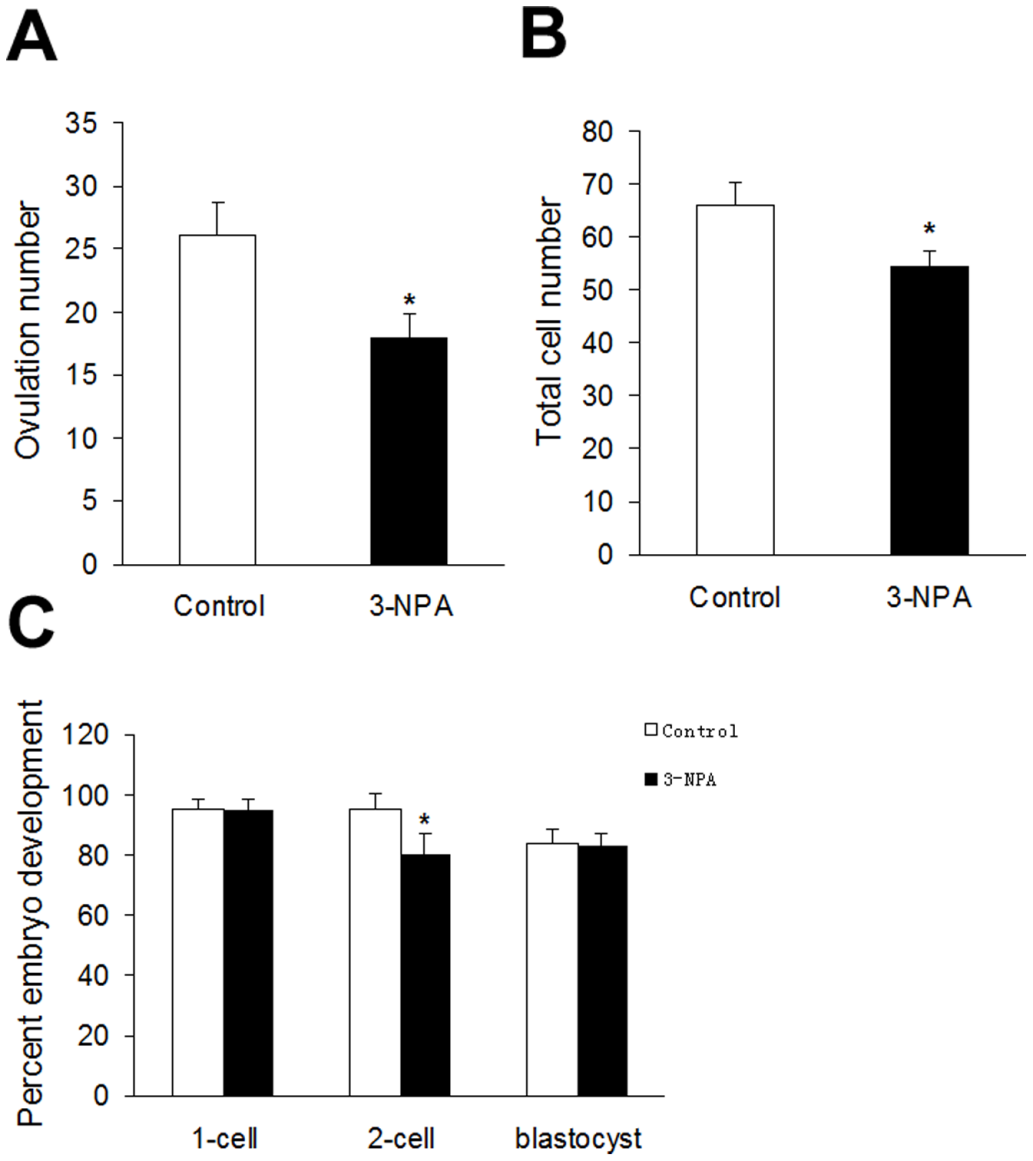
Effect of oxidative stress on ovulation number and early embryonic development. The effect of oxidative stress on ovulation number (A), total cell number of blastocyst cells (B), and oocyte developmental competence (C). Values are expressed as the percentage of zygotes developed to each stage. Data are presented as mean ± SEM per female; * P<0.05 versus control.

Because administration of 3-NPA (12.5 mg/kg) induced apoptosis in granulosa cells, we tested its effect on ovulation number and zygote development. Eggs were isolated from the oviducts of 3-NPA and control mice by intraperitoneal administration of PMSG and hCG. Fertilization rates and the percentage of development to blastocyst were unaffected by the treatment. However, the cleavage rate, number of oocytes released, and number of blastocyst cells decreased significantly (Figure 6). DAPI staining showed that total cell numbers decreased significantly in blastocysts developed from oocytes that were obtained from mice treated with 3-NPA (12.5 mg/kg), compared to the control mice.

### Effect of 3-NPA on the levels of reproductive hormones

**Figure 7:**
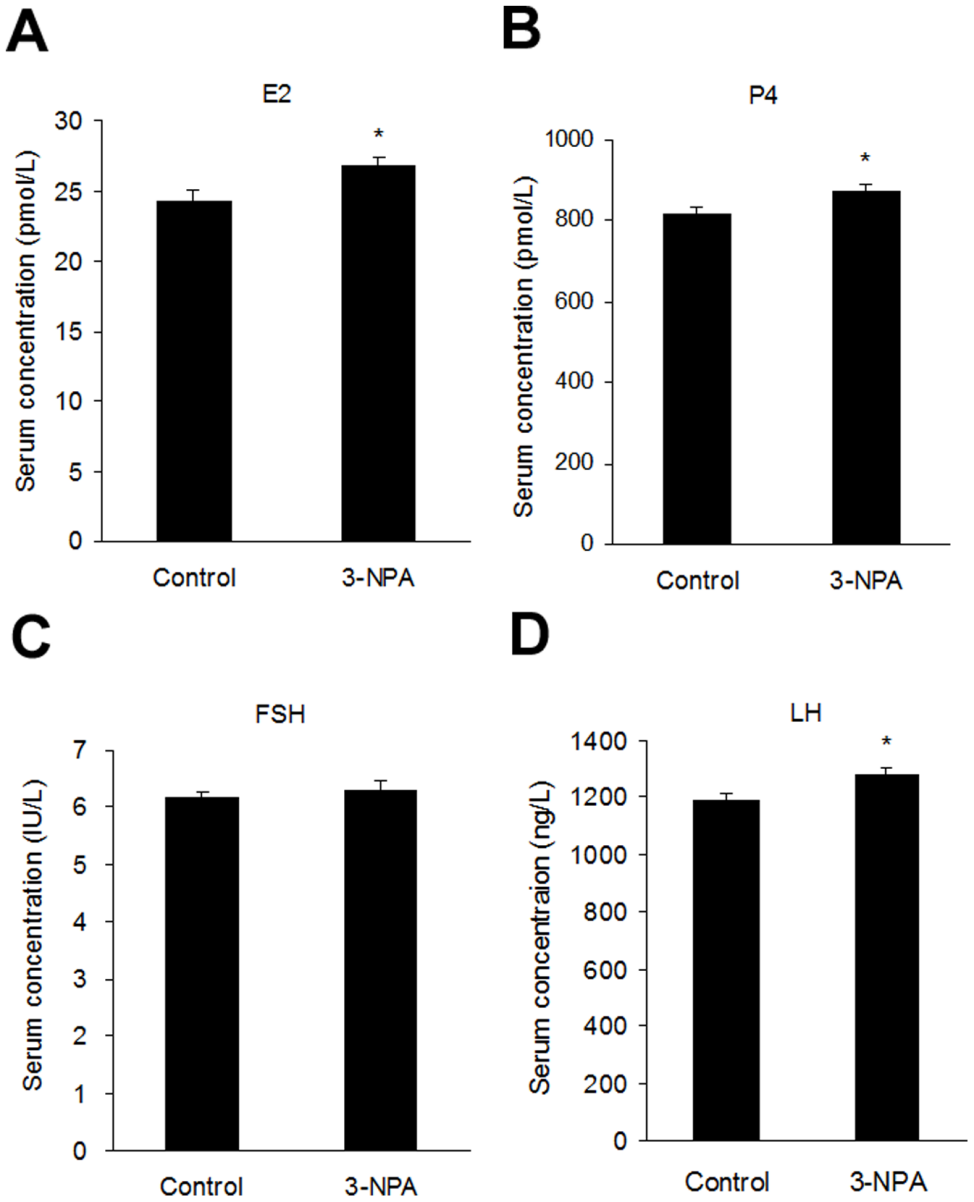
Serum levels of reproductive hormones in 3-NPA-treated mice. The serum levels of estradiol (E2) (A), progesterone (P4) (B), follicle-stimulating hormone (FSH) (C) and luteinizing hormone (LH) (D) were determined by ELISA in control and 3-NPA-treated mice. Data show the means ± SEM (n = 10). *P<0.05, compared to control.

Serum levels of E2, P4 and LH increased significantly in 3-NPA-treated animals compared with control group (Figure 7, A, B and D). No difference was observed in FSH serum levels of the 3-NPA versus control groups (Figure 7C).

### Effect of 3-NPA-induced ovarian oxidative stress on reproductive ability

**Figure 8:**
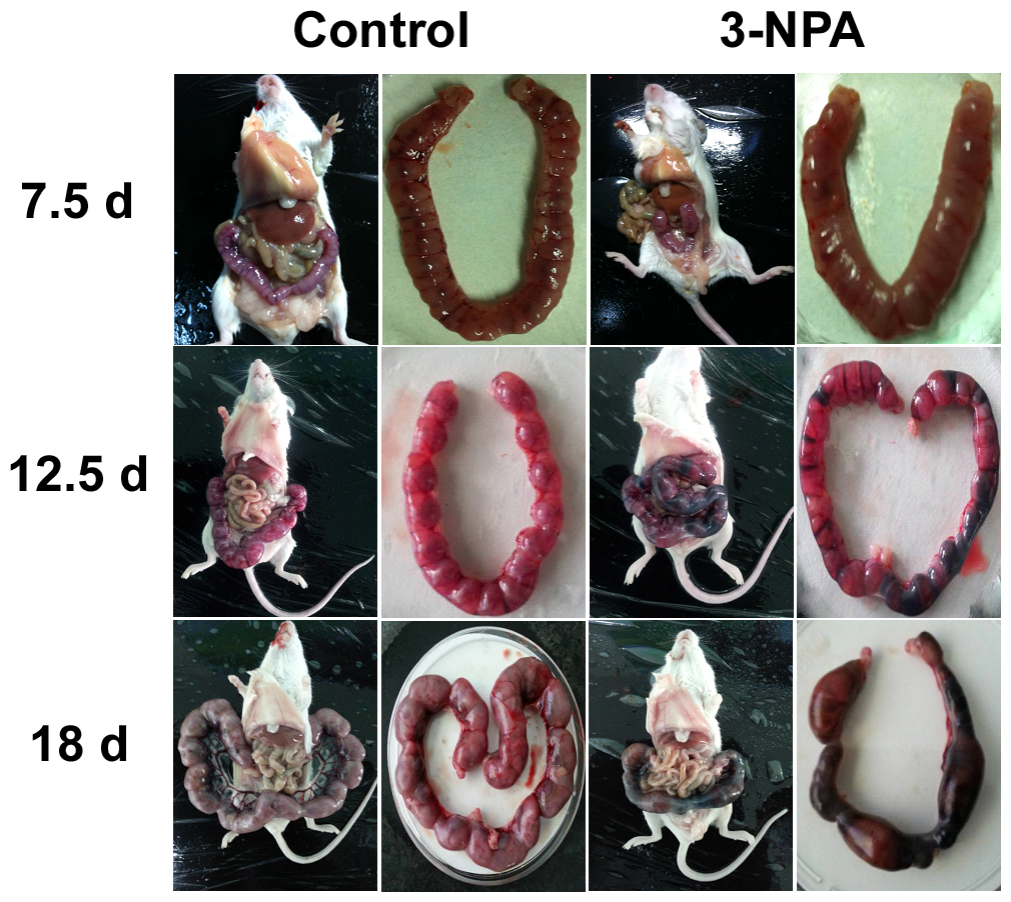
Effects of 3-NPA treatment on fetus/embryo development in vivo. Female mice were intraperitoneally injected with saline or oxidant 3-NPA for 7 days, respectively. On GD7.5, GD12.5 and GD18, the pregnant mice (n = 5, each group) were sacrificed and mouse embryos were isolated. The growth development of embryos was observed.

The occurrence of vaginal plugs and pregnancies was significantly lower in the 3-NPA group compared to control mice (reproductive outcome data are summarized in Table 1). In addition, the number of fetal mice at GD 7.5, 12.5 and 18 in the 3-NPA group were significantly decreased compared to in control mice. At the same gestational stages, the 3-NPA treatment in vivo retarded fetal growth and increased the frequency of embryo resorption and fetal deaths (Figure 8).

**Table 1:** Effect of 3-NPA-induced ovarian oxidative stress on reproductive ability.

Group	NO. of mice	NO. of vaginal plug (%)	NO. of Pregnancy (%)	Average number of fetal mice		
				7.5 d	12.5 d	18 d
Control	36	26 (72.2)	20(76.9)	24.33±0.64	23.50±0.76	18.50±0.32
3-NPA	45	24 (53.3)*	16(66.7)*	16.50±0.58*	15.25±0.52*	13.50±0.37*

Data are presented as mean ± SEM.

* *P*<0.05 compared to Control;

Abbreviations: 3-NPA, 3-nitropropionic acid; NO., Number.

### Effect of 3-NPA on neurobehavioral exhibition

Figure S1A shows the effect of pretreatment of mice with 3-NPA (12.5 mg/kg) on immobility time in the tail suspension test. Results showed that 3-NPA (12.5 mg/kg) treated mice did not have different immobility times throughout the experiment compared with control animals. Lack of motor incoordination and the balance of mice were assessed with the rotarod test. Figure S1B shows the effect of pretreatment of mice with 3-NPA (12.5 mg/kg) on the rotarod activity in the rotarod test. Results showed that 3-NPA (12.5 mg/kg) treated mice did not show any effect in rotarod activity and did not have different fall-off time throughout the experiment compared with control animals. Figure S1C shows the effect of pretreatment of mice with 3-NPA (12.5 mg/kg) on the immobility time in the forced swimming test. Results showed that female mice treated with 3-NPA (12.5 mg/kg) did not show a marked behavioral despair, with no difference in immobility time when submitted to forced swimming.

### Effect of 3-NPA on the telomerase activity and telomere length of organ tissues

To study the effect of 3-NPA on the telomerase activity and telomere length of organ tissues, telomerase activity and telomere length were measured in different tissues from control and 3-NPA treatment animals. Our results showed that treatment had no effect on the telomerase activity of brain, liver, spleen, kidney and ovary tissues (Figure S2A). The telomere length was significantly lower in liver tissue from 3-NPA-treated animals compared with control animals. The shortened telomere length was also observed in ovary in 3-NPA-treated animals, but there was no significant difference between control and 3-NPA groups (Figure S2B).

## Discussion

In this study, we used 3-NPA to establish a mouse model for simulating the effects of oxidative stress on mammalian ovaries. Our data showed that ROS levels significantly increased in follicular granulosa cells and other parts of the ovary, but not in other tissues (brain, liver, kidney and spleen) when mice were injected with low doses (12.5 mg/kg, for 7 days) of 3-NPA. This study demonstrated a clear impact of oxidative stress on female reproduction. Our study suggests that this model has important similarities with the ovarian pathological condition typically induced by oxidative stress [38], [39]. To our knowledge, this is the first work to show that 3-NPA reduces the number of large follicles, impairs oocyte development, and increases the percentage of atretic large follicles and ROS levels in oocytes and granulosa cells.

Although there are many existing models of oxidative stress, few are well suited to studies of ovarian oxidative stress. Methoxychlor (MXC) is an organochlorine pesticide, which can specifically induce atresia of antral follicles in vivo, and inhibit growth and increase atresia of antral follicles in vitro, through an oxidative stress pathway in mice [40]. However, MXC can result in liver and kidney damage, immune dysfunction and disturbed hormonal balance [41]. The heavy metal lead (Pb) can affect the follicular development and maturation, and increase the number of atretic antral follicles, but the main targets of Pb toxicity are the red blood cell, the central and peripheral nervous system and the kidney [42]. Cigarette smoke can reduce the number of primordial follicles, but it can also cause lung disease and cancer [43], [44]. Although 4-Vinylcyclohexene can damage follicular development capacity, it is not related to oxidative stress [3]. Oxidative stress has been reported when rodent models were treated with 3-NPA [19], [20], [45]. However, there is little evidence of the effects of oxidative stress effects on follicular development and atresia in rodent models. Our previous studies have demonstrated the effect of 3-NPA on ovaries, which showed that mice treated with higher doses of 3-NPA (twice daily for 5 days at a dose of 50 mg/kg) had significantly higher ROS levels in follicular granulosa cells but not other tissues, compared to controls [13]. This study also show that the low doses of 3-NPA (12.5 or 25 mg/kg) treatment significantly reduced large follicle numbers and increased follicular atresia in the mouse ovaries. These findings are consistent with previous reports of oxidative stress-induced follicular atresia in other mammals treated with oxidants [46], [47].

In a well-developed follicle, ROS and antioxidants remain in balance. Oxidative stress occurs when this delicate balance between ROS and antioxidants is disrupted, resulting in apoptosis of granulosa cells, oocytes or embryos [5], [48], [49]. Apoptosis is a process that can be triggered by numerous factors, including oxidative stress [50]. It has been reported that heavy metals, pesticides and other environmental toxins may cause apoptosis of granulosa cells and follicular atresia through an oxidative stress pathway [2], [40], [42], [51]. Many studies have shown that granulosa cell apoptosis induces follicular atresia, reduces the number of oocytes and damages their quality [52]–[55]. It may also lead to the loss of non-dominant follicles through atresia [56], [57]. Our results further demonstrated that the number of oocytes produced had a negative correlation with the incidence of granulosa cells apoptosis. We reasoned that granulosa cells apoptosis likely resulted in follicular atresia and reduced the number of ovulated oocytes.

Several studies have shown that oxidative stress can reduce the number of follicle and oocytes. For example, one study revealed that oxidative stress causes a significant decrease in the number of follicles and the ovulated oocytes during repeated ovulation [58]. Exposure to agents known to cause oxidative stress, such as ionizing radiation, heavy metals, tobacco smoke or polycyclic aromatic hydrocarbons (PAHs) causes rapid follicle loss [59]. In vivo studies have shown that ionizing radiation can destroy small follicles and antral follicles in mice [60], [61]. Female offspring at postnatal day (PND) 21, born to the lactating mother rats that have been treated with chromium (200 mg/L) in drinking water, have significantly fewer follicles [62]. The ovaries of offspring born to female mice exposed to PAHs contained only a third of the ovarian follicle pool compared with offspring to unexposed females [59]. Our current study also shows that ovarian oxidative stress caused by 3-NPA treatment can significantly reduce the number of large follicle and the ovulated oocytes.

Only high quality oocytes can produce well-developed embryos. Oocyte quality is very important for determining the quality of the early stages of embryo development. However, excessive amount of ROS production in oocytes and embryos causes oxidative stress and impairs oocyte and embryo quality. Many studies have shown that ROS reduce oocyte and embryo quality [5], [7]. Oxidative stress in mural granulosa cells and cumulus granulosa cells induced by ROS reduces fertilization rates and subsequently leads to a decrease in the quality of embryos [63]. When preovulatory follicles from mice were incubated with H_2_O_2_, the percentage of mature oocytes with a first polar body was significantly reduced [64]. There is also evidence that ROS have been implicated in the arrest of mammalian embryo development, in vitro. In mouse embryo development, the two-cell embryo block was observed, which is associated with a rise in ROS [65]. In this study, we also found that mice treated with 3-NPA (12.5 mg/kg) have significantly higher ROS production in granulosa cells and oocytes, fewer oocytes, and lower developmental competence in embryos.

Follicular components—such as cumulus cells, granulosa cells and follicular fluid—may protect oocytes from the damaging effects of oxidative stress [66], [67]. It is well known that endogenous antioxidant enzymes like GP_X_, SOD, and CAT, along with non-enzymatic antioxidants in the follicles, work to neutralize ROS and protect the oocyte and embryo from damage [68]–[70]. Our study shows that 3-NPA significantly increases the activity of antioxidant enzymes in ovarian tissues and granulosa cells. We inferred that the increases in enzyme activity in response to 3-NPA may be an early response of organism to protect the follicles from oxidative stress damages. While no studies have reported the effect of 3-NPA on antioxidant enzymes in the ovary, some have shown that 3-NPA affects antioxidant enzymes in other tissues. For example, 3-NPA caused significant increase in SOD activity in the striatum of rats dosed with 3-NPA, and this change could be prevented by prior administration of melatonin [20]. Another study indicated that the activities of SOD and GP_X_ increased significantly in liver of rats dosed with 80 mg/kg 3-NPA. Our study supports and broadens the conclusion of those findings.

In conclusion, we have established a novel animal model of ovarian oxidative stress induced by 3-NPA intraperitoneal injection. Our approach provides a useful platform for further investigation of the effects of ovarian oxidative stress induced by ROS on oocyte and follicle development, and for the screening of new antioxidants that could be more effective in preventing ovarian oxidative damage.

## Supporting Information

Figure S1 **Effect of pretreatment with 3-NPA on neurobehavioral test results.** Mice were intraperitoneally injected with saline or oxidant 3-NPA. After treatment for 7 days, the neurobehavior of subjects was assessed. (A) Immobility time in the tail suspension test was scored for a 4 min period. (B) Fall off time in the rod rotarod test, with the length of time on the rod used as the measure of competency. (C) Immobility time in the forced swimming test, the duration of immobility during the last 4 min was measured. Values are expressed as mean ± S.E.M. (n = 10) *P<0.05 versus the saline-treated group.
(TIF)

Figure S2 **Comparison of telomerase activity and telomere length between 3-NPA and control groups.** Mice were intraperitoneally injected with saline or 3-NPA (12.5 mg/kg) for 7 days, Organ tissues were collected for the measurement of telomerase activity and telomere length. (A) Telomerase activity was assessed using a Telomerase ELISA kit. (B) Telomere length was analyzed by a real-time PCR. The means of the 2 groups were compared using a t-test. Values are expressed as mean ± S.E.M. (n = 5) *P<0.05 versus the saline-treated group.
(TIF)

Table S1 **Primer sequences for real-time RT-PCR.**
(DOC)
