# Supplementary figures and images for: 3-Nitropropionic Acid Induces Ovarian Oxidative Stress and Impairs Follicle in Mouse
Source: PLoS One. 2014 Feb 5;9(2):e86589. doi: 10.1371/journal.pone.0086589 (PMC3914797; doi:10.1371/journal.pone.0086589)

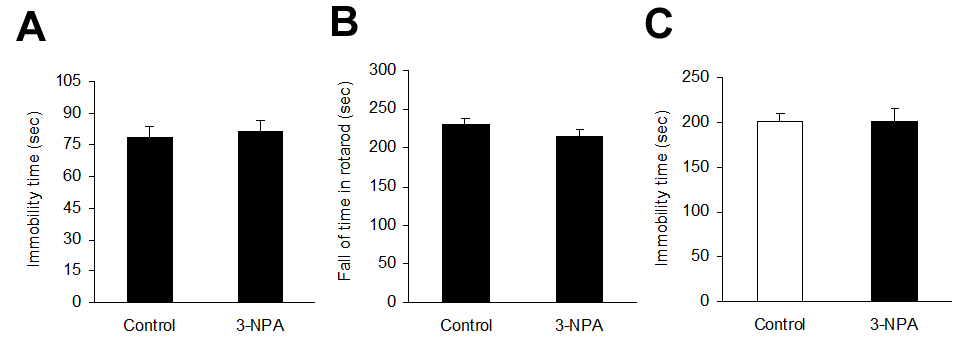

Supplement: Figure S1 — Effect of pretreatment with 3-NPA on neurobehavioral test results. Mice were intraperitoneally injected with saline or oxidant 3-NPA. After treatment for 7 days, the neurobehavior of subjects was assessed. (A) Immobility time in the tail suspension test was scored for a 4 min period. (B) Fall off time in the rod rotarod test, with the length of time on the rod used as the measure of competency. (C) Immobility time in the forced swimming test, the duration of immobility during the last 4 min was measured. Values are expressed as mean ± S.E.M. (n = 10) *P<0.05 versus the saline-treated group. (TIF) [file pone.0086589.s001.tif]

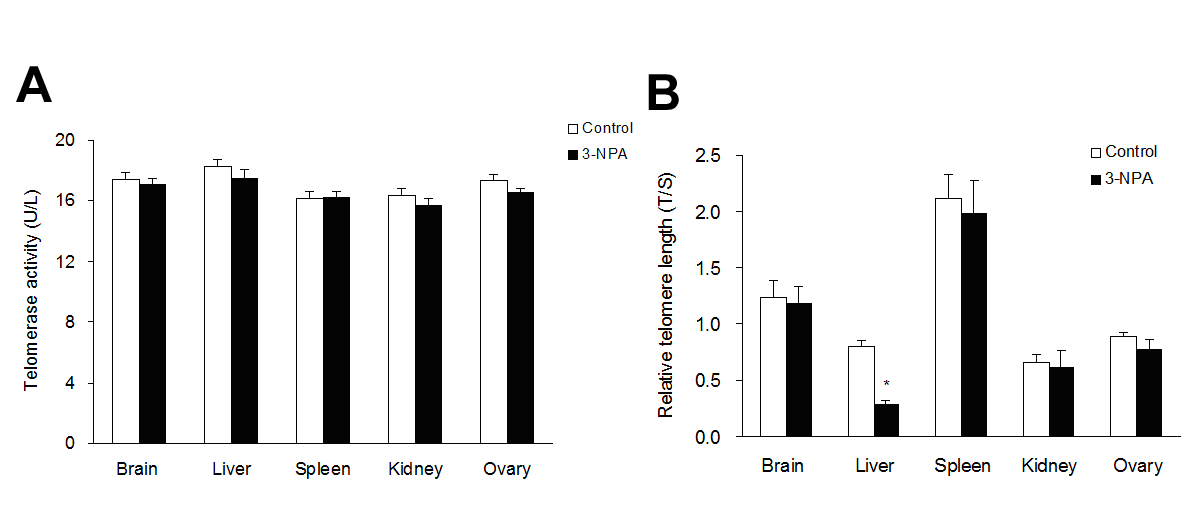

Supplement: Figure S2 — Comparison of telomerase activity and telomere length between 3-NPA and control groups. Mice were intraperitoneally injected with saline or 3-NPA (12.5 mg/kg) for 7 days, Organ tissues were collected for the measurement of telomerase activity and telomere length. (A) Telomerase activity was assessed using a Telomerase ELISA kit. (B) Telomere length was analyzed by a real-time PCR. The means of the 2 groups were compared using a t-test. Values are expressed as mean ± S.E.M. (n = 5) *P<0.05 versus the saline-treated group. (TIF) [file pone.0086589.s002.tif]
