# Supplementary material for: 3-Nitropropionic Acid Induces Ovarian Oxidative Stress and Impairs Follicle in Mouse
Source: PLoS One. 2014 Feb 5;9(2):e86589. doi: 10.1371/journal.pone.0086589 (PMC3914797; doi:10.1371/journal.pone.0086589)
Supplement: Table S1 — Primer sequences for real-time RT-PCR. (DOC) [file pone.0086589.s003.doc]

| Gene | Forward Primer | Reverse Primer | Base pairs | T | Accession Number |
| --- | --- | --- | --- | --- | --- |
| GAPDH | ACCACAGTCCATGCCATCAC | TCCACCACCCTGTTGCTGTA | 452 | 56 | M32599 |
| Bax | CCAGGATGCGTCCACCAAGA | GGTGAGGACTCCAGCCACAA | 394 | 57 | NM_007527.3 |
| Bcl-2 | GTGGATGACTGAGTACCTGAACC | AGCCAGGAGAAATCAAACAGAG | 120 | 60 | NM_009741.3 |
| SOD2 | ATGGTGGGGGACATATT | GAACCTTGGACTCCCACAGA | 166 | 57 | NM_017051 |
| CAT | CCTCGTTCAGGATGTGGTTT | TCTGGTGATATCGTGGGTGA | 130 | 60 | NM_009804.1 |
| GPx | GTCCACCGTGTATGCCTTCT | TCTGCAGATCGTTCATCTCG | 152 | 57 | NM_008160 |

Table S1. Primer sequences for real-time RT-PCR

Abbreviations: GAPDH, glyceraldehyde-3-phosphate dehydrogenase; Bax, Bcl-2 associated X protein; Bcl-2, B-cell leukemia/lymphoma 2; SOD2, manganese superoxide dismutase; CAT, catalase; GPx, glutathione peroxidase; Base pairs, the product length and T, the annealing temperature given as °C.
